# Potentialities of CdZnTe Quasi-Hemispherical Detectors for Hard X-ray Spectroscopy of Kaonic Atoms at the DAΦNE Collider

**DOI:** 10.3390/s23177328

**Published:** 2023-08-22

**Authors:** Leonardo Abbene, Antonino Buttacavoli, Fabio Principato, Gaetano Gerardi, Manuele Bettelli, Andrea Zappettini, Massimiliano Bazzi, Mario Bragadireanu, Michael Cargnelli, Marco Carminati, Alberto Clozza, Griseld Deda, Raffaele Del Grande, Luca De Paolis, Laura Fabbietti, Carlo Fiorini, Carlo Guaraldo, Mihail Iliescu, Misahiko Iwasaki, Aleksander Khreptak, Simone Manti, Johann Marton, Marco Miliucci, Pawel Moskal, Fabrizio Napolitano, Szymon Niedźwiecki, Hiroaky Ohnishi, Kristian Piscicchia, Yuta Sada, Francesco Sgaramella, Hexi Shi, Michalł Silarski, Diana Laura Sirghi, Florin Sirghi, Magdalena Skurzok, Antonio Spallone, Kairo Toho, Marlene Tüchler, Oton Vazquez Doce, Chihiro Yoshida, Johannes Zmeskal, Alessandro Scordo, Catalina Curceanu

**Affiliations:** 1 Department of Physics and Chemistry (DiFC)—Emilio Segrè, University of Palermo, Viale delle Scienze, Edificio 18, 90128 Palermo, Italy; 2 Laboratori Nazionali di Frascati, INFN, Via E. Fermi 54, 00044 Frascati, Italycatalina.curceanu@lnf.infn.it (C.C.); 3 Istituto Materiali per l’Elettronica e il Magnetismo, Consiglio Nazionale delle Ricerche (IMEM/CNR), Parco Area delle Scienze 37/A, 43100 Parma, Italy; 4 Horia Hulubei National Institute of Physics and Nuclear Engineering, Str. Atomistilor No. 407, 077125 Măgurele, Romania; 5 Stefan-Meyer-Institut für Subatomare Physik, 1030 Vienna, Austria; 6 Politecnico di Milano, Dipartimento di Elettronica, Informazione e Bioingegneria, 20133 Milano, Italy; 7 Istituto Nazionale di Fisica Nucleare (INFN), Sezione di Milano, 20133 Milano, Italy; 8 Physik Department E62, Technische Universität Münnchen, 85748 Garching, Germany; 9 Institute of Physical and Chemical Research (RIKEN), Wako, Tokyo 351-0198, Japan; 10 Faculty of Physics, Astronomy and Applied Computer Science, Jagiellonian University, 30-348 Krakow, Poland; 11 Center for Theranostics, Jagiellonian University, Kopernika 40, 31-501 Krakow, Poland; 12 Research Center for Electron Photon Science (ELPH), Tohoku University, Sendai 982-0826, Japan; 13 Centro Ricerche Enrico Fermi—Museo Storico della Fisica e Centro Studi e Ricerche “Enrico Fermi”, 00184 Roma, Italy; 14 Vienna Doctoral School in Physics, University of Vienna, 1090 Vienna, Austria

**Keywords:** CdZnTe detectors, quasi-hemispherical detectors, compound semiconductor detectors, hard X-ray spectroscopy, kaonic atoms, digital pulse processing electronics

## Abstract

Kaonic atom X-ray spectroscopy is a consolidated technique for investigations on the physics of strong kaon–nucleus/nucleon interaction. Several experiments have been conducted regarding the measurement of soft X-ray emission (<20 keV) from light kaonic atoms (hydrogen, deuterium, and helium). Currently, there have been new research activities within the framework of the SIDDHARTA-2 experiment and EXCALIBUR proposal focusing on performing precise and accurate measurements of hard X-rays (>20 keV) from intermediate kaonic atoms (carbon, aluminum, and sulfur). In this context, we investigated cadmium–zinc–telluride (CdZnTe or CZT) detectors, which have recently demonstrated high-resolution capabilities for hard X-ray and gamma-ray detection. A demonstrator prototype based on a new cadmium–zinc–telluride quasi-hemispherical detector and custom digital pulse processing electronics was developed. The detector covered a detection area of 1 cm^2^ with a single readout channel and interesting room-temperature performance with energy resolution of 4.4% (2.6 keV), 3% (3.7 keV), and 1.4% (9.3 keV) FWHM at 59.5, 122.1, and 662 keV, respectively. The results from X-ray measurements at the DAΦNE collider at the INFN National Laboratories of Frascati (Italy) are also presented with particular attention to the effects and rejection of electromagnetic and hadronic background.

## 1. Introduction

Kaonic atoms are formed when K^−^ mesons are stopped and captured by atoms. The K^−^ replaces an electron in an excited atomic level, producing an electromagnetic cascade process. In this case, the strong interaction between the kaon and the atomic nucleus/nucleons induces an energy level shift and broadening with respect to the purely electromagnetic values [1,2,3]. Kaonic atoms represent an interesting tool to study the low-energy regime of quantum chromodynamics (QCD). Due to the much heavier mass of K^−^ compared to e^−^, the lower levels are close enough to the nucleus to be influenced by the strong short-range interaction between K^−^ and the nucleus/nucleons [1,2,3]. 

X-ray spectroscopy is a powerful technique to investigate the energy shift and width of the levels resulting from the strong kaon–nucleus/nucleon interaction. Despite intense research activities on kaonic atom X-ray measurements in the 1970s and 1980s [4,5,6], more accurate and precise measurements are necessary to better study kaon–nucleus/nucleon interactions. From the 2000s, key results have been obtained within DEAR (DAΦNE Exotic Atoms Research) [7], SIDDHARTA (Silicon Drift Detectors for Hadronic Atom Research by Timing Application), [8] and SIDDHARTA-2 [9] experiments at the DAΦNE (Double Annular Φ Factory for Nice Experiments) collider at the INFN National Laboratories of Frascati (Italy). The DAΦNE electron–positron collider is an ideal facility for kaonic atom physics that is able to produce low-momentum (∼127 MeV/c) and quasi-monoenergetic kaons through the decay of the *ϕ* resonance from electron–positron annihilation. It allows investigations on the strong interaction in the strangeness sector at low energies. These experiments mainly focus on precise measurements of low-energy X-ray emission (<20 KeV) from light kaonic atoms (hydrogen, deuterium, and helium). In this case, silicon drift detectors (SDD), invented in 1984 by Gatti and Rehak [10,11], are used, representing the best solution for up to 20 keV [12,13].

Recently, within the framework of the SIDDHARTA-2 experiment and EXCALIBUR proposal (EXtensive Kaonic Atoms research: from LIthium and Beryllium to URanium) [14], there have been new research activities aimed at measuring high-energy X-ray transitions from intermediate kaonic atoms, such as carbon, aluminum, and sulfur, with expected X-ray energies between 20 and 300 keV. Concerning the X-ray detection process, new detection systems should be used with higher detection efficiency than the silicon ones. Cadmium zinc telluride (CdZnTe or CZT) detectors represent a valid solution for high-resolution measurements of hard X-rays from intermediate kaonic atoms (>20 keV). The combination of high atomic number (Z_max_ = 52) and wide bandgap (~1.6 eV), together with the continuous progress in crystal growth and device technology, potentially allows high detection efficiency within a few millimeters and excellent room-temperature performance [15,16,17,18,19,20,21,22,23,24]. CZT detectors with complex pixel and strip electrode layouts have been widely proposed and developed for X-ray and gamma-ray spectroscopy and imaging, mainly focusing on medical applications [25], nuclear security [18], and food/pharma inspections [26,27,28,29]. Few experiments have proposed their use in nuclear and particle physics [30]. In the context of kaonic atom X-ray spectroscopy, the nonimaging requirements of these measurements open up the possibility of using simple detector layouts with a moderate number of readout channels. CZT coplanar-grid [31,32,33] and quasi-hemispherical [34,35,36,37,38] detectors represent interesting detector layouts for these applications, allowing a large detection area (>10 × 10 mm^2^ for each detector) with a moderate number of readout channels. Moreover, the electron-sensing properties of these detectors [38,39] are also able to minimize the effects of poor hole transport properties in the spectroscopic performance (incomplete charge collection). The electron-sensing properties are due to the shape of the weighting field/potential, which results in the signals mainly being characterized by electron motion [35,39]. In comparison with coplanar-grid detectors, the quasi-hemispherical layout also allows lower electronic noise (due to the low electrode capacitance), does not require analytical operations between the pulses and the electrodes, and therefore achieves better energy resolution at low energies (<100 keV) [38]. Besides the detector, the electronics also play a key role, strongly influencing the detector response and temporal coincidence with external signals for background reduction. Nowadays, digital pulse processing (DPP) electronics have been shown to have better performance than the analog ones, allowing flexible and dedicated analysis for performance improvements [40,41,42,43,44,45]. The digital approach, based on the direct digitization of the output pulses from the detector/preamplifier, gives high timing resolution and the possibility to implement new processing procedures, which are challenging to realize through the analog approach. 

In this work, we present the performance of a new quasi-hemispherical CZT-based detection system for hard X-ray spectroscopy of kaonic atoms. The prototype, as a demonstrator module of a more complex system, is based on a new CZT quasi-hemispherical detector developed at IMEM-CNR of Parma and digital electronics developed at DiFC of the University of Palermo (Palermo, Italy). It is able to perform pulse shape and height analysis of the detector channels and temporal coincidences with external triggers for background reduction. Firstly, we present the results from spectroscopic and timing characterization of the detection system with laboratory calibration sources. Then, hard X-ray spectra measurements under the DAΦNE collider environment are presented with particular attention to reduction of the electromagnetic and hadronic background. 

## 2. Materials and Methods 

### 2.1. The CZT Quasi-Hemispherical Detector

**Figure 1:**
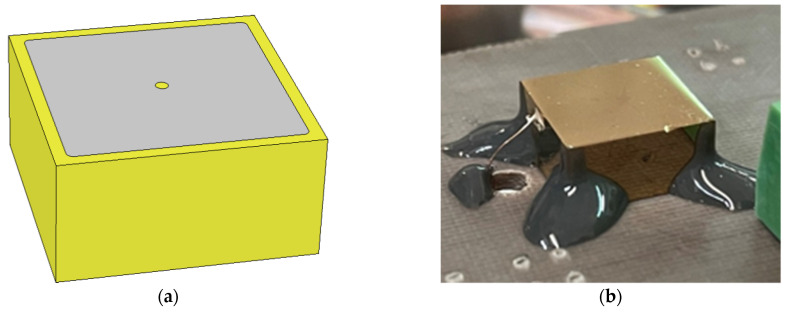
(**a**) The electrode layout of the CZT quasi-hemispherical detector (10 × 10 × 5 mm^3^). The circular pixel in the anode side is characterized by a diameter of 750 µm. (**b**) A picture of the detector from the cathode side.

Figure 1 shows the anode layout of the CZT quasi-hemispherical detector and a picture of the detector (cathode side view) wire-bonded on the readout board. The detector was fabricated at IMEM-CNR of Parma (Parma, Italy) in collaboration with due2lab company (Reggio Emilia, Italy). It is based on a LF (low-flux) THM (traveling heater method)-CZT crystal (10 × 10 × 5 mm^3^) provided by Redlen Technologies (Saanichton, BC, Canada). 

**Figure 2:**
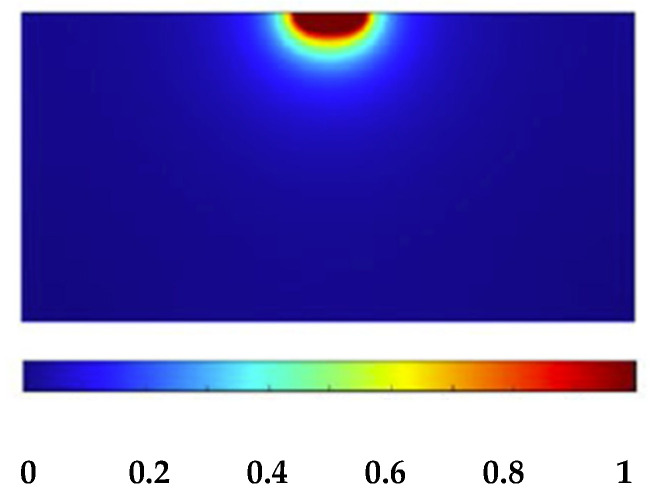
Calculated weighting field [39]. The weighting field is more concentrated near the anode pixel, confirming the electron-sensing properties of the detector.

**Figure 3:**
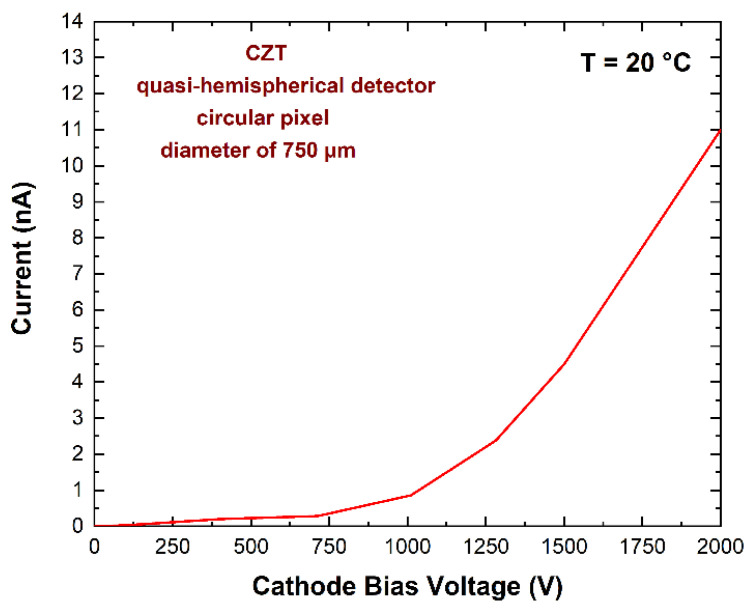
Measured I–V curve of the anode pixel of the CZT quasi-hemispherical detector.

THM-CZT crystals with high electron mobility–lifetime (μ_e_τ_e_ > 10^−2^ cm^2^/V), termed LF-CZT, are routinely produced by Redlen Technologies for the development of thick electron-sensing detectors for low-flux gamma-ray measurements [15,46,47,48,49]. Gold electroless contacts have been realized for both cathode and anode electrodes. Recently, very-low-noise gold contacts were developed on CZT detectors at IMEM-CNR of Parma [49,50,51,52], ensuring low leakage currents at room temperature (<5 nA cm^−2^ at 1000 V cm^−1^). Regarding the anode layout, a circular pixel is present with a diameter of 750 µm, properly selected after simulations and experimental measurements [35]. This pixel size represents the best compromise between the reduction of electronic noise and enhancements in the charge collection properties. This electrode layout confers electron-sensing properties to the detector, in agreement with the behavior of the weighting field. It is calculated and reported in Figure 2. The calculus of the weighting field is based on the finite elements method (FEM) through COMSOL Multiphysics [39]. Following the Shockley–Ramo theorem [53], the induced current and charge on the electrodes are related to the weighting field and weighting potential, respectively. The weighting field is more concentrated near the anode pixel, and the induced current therefore has more contribution from the electrons. The hole contribution is only present in the signals related to photon interactions near the anode pixel. The results from electrical measurements (Figure 3) showed low leakage current values at room temperature, less than 1 nA at −1000 V. The current–voltage (I–V) curve was measured with a Keithley 2635B instrument (Keithley Instruments LLC, Solon, OH, USA) configured as an electrometer and CAEN NDT1471 (CAEN S.p.A., Viareggio, Italy) as a bias voltage supplier. This result demonstrates the high quality of our gold electroless contact process for these detectors. 

**Figure 4:**
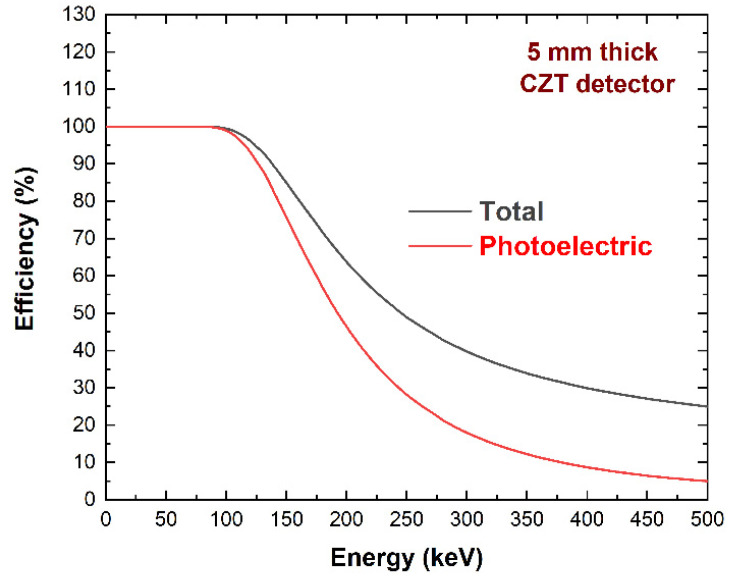
Calculated detection efficiency of 5 mm CZT material thickness at various X-ray energies.

As shown in Figure 4, the thickness of 5 mm ensured good detection efficiency at the expected X-ray energies (20–300 keV).

### 2.2. Readout Electronics and Pulse Processing

**Figure 5:**
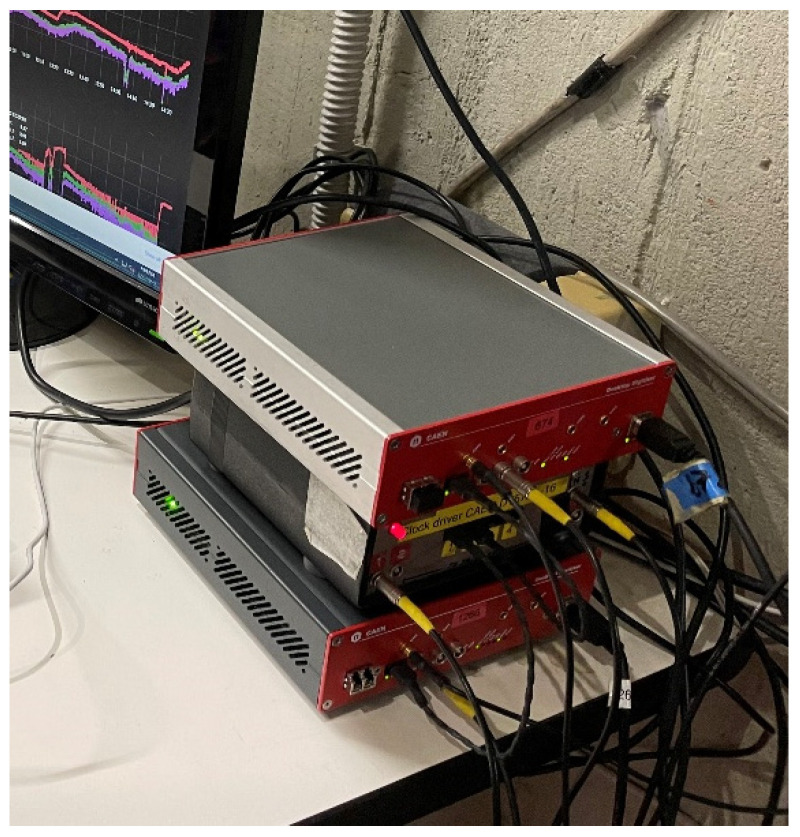
The 8-channel digital pulse processing (DPP) electronic device. The digital system is based on two digitizers and an external clock. Pulse processing was performed by a custom firmware [54,55].

Read-out electronics are based on analog charge-sensitive preamplifiers (CSPs) and digital pulse processing (DPP) electronics. The two channels of the detector (anode and cathode electrodes) were AC coupled to hybrid CSPs and processed by an 8-channel DPP electronic device. The CSPs and the digital electronics were developed at DiFC of the University of Palermo (Italy). The preamplifiers are characterized by an equivalent noise charge (ENC) of about 100 electrons (equivalent to about 1 keV FWHM for CZT detectors) and equipped with a resistive-feedback circuit with exponential decay and time constant of 100 µs. The digital electronic device consists of 2 commercial digitizers with 8 channels (DT5724, 16 bit, 100 MS/s, CAEN S.p.A., Italy), driven by an original firmware developed by our group [54,55]. A picture of the digital electronic device installed in the SIDDHARTA-2 control room (outside the DAΦNE collider) is shown in Figure 5. An external clock was used to synchronize the digitizers. The digitizers are driven by a PC, where both the acquisition and the analysis can be controlled. Digital electronics are able to do both on-line and off-line pulse processing. In our case, the digital system performed on-line pulse detection and off-line pulse shape and height analysis (PSHA). The off-line PSHA was applied on a sequence of selected CSP pulses (snapshot waveform) with the related on-line measured arrival times.

The details of *on-line* pulse processing are described below:

(i) *Pulse detection*: The output waveforms from each detector–preamplifier are shaped using the classical single delay line (SDL) shaping technique [56], i.e., using a differentiation operation in the time domain; the time width of the SDL pulses (equal to the sum of the peaking time and the delay time) represents the dead time of the detection process (paralyzable dead time) [56]. Typically, the delay of the SDL shaping is selected with values less than the peaking time of the CSP pulses in order to reduce the dead time. The arrival time of each event is calculated through the ARC (amplitude and rise time compensation) timing marker (at the leading edge of the SDL pulses), necessary to reduce the effects of time jitters and amplitude/rise time walks [56]. The timing resolution is less than 10 ns.
(ii) *Snapshot waveforms (SWs)*: They consist of waveforms of CSP output pulses with the related arrival times produced for *off-line* PSHA. Each CSP pulse is centered on a time window, the duration of which is termed *snapshot time* (*ST*). A pulse is accepted if it is not preceded and not followed by another pulse in the *ST/2* time windows. The *ST* parameter can be selected by the user.

**Figure 6:**
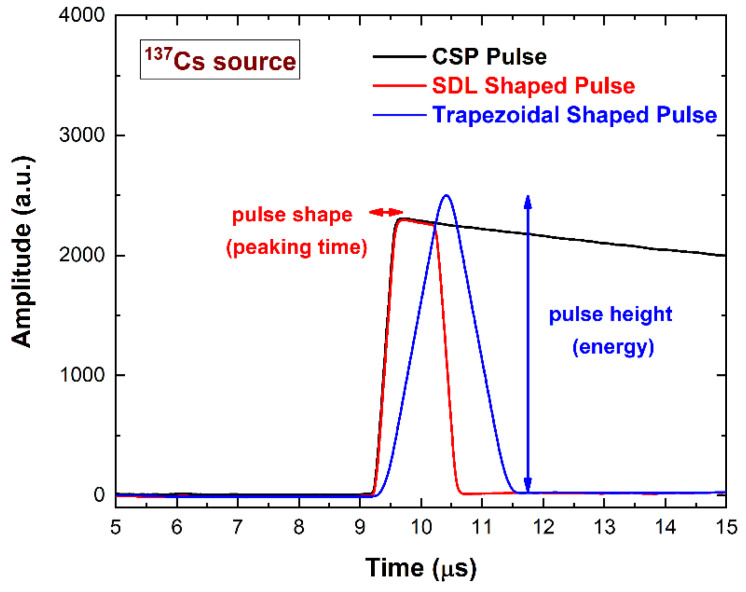
Measured output anode pulse from the charge sensitive preamplifier (CSP) after digitization (black line), after SDL shaping (red line) with a delay line of 1000 ns, and after trapezoidal filtering (blue line). The CSP output pulse is within a snapshot time (*ST*) of 10 µs.

The CSP output pulses of the SWs are *off-line* processed with dedicated PSHA. The height (i.e., the photon energy) and the peaking time (i.e., the pulse shape) of the pulses are calculated after SDL and trapezoidal shaping, as shown in Figure 6. In this case, the delay of the SDL shaping (acting as the shaping time constant of a shaping amplifier) is always selected to be longer than the peaking time of the CSP pulses, thus avoiding ballistic deficit effects [57]. To minimize the degradation of the spectroscopic response at different input counting rates (ICRs), a baseline recovery was implemented using the running average of a fixed number of samples preceding each pulse. We used two custom software for acquisition and analysis. A C++ based program was developed to set the parameters of the digitizer (acquisition time, channels, etc.) for the acquisition of the SWs. We used a snapshot time (*ST*) of 10 µs. Another program (MATLAB) was developed to analyze the pulses of the SWs and generate the energy spectra. The energy resolution was estimated using a dedicated best-fitting function that also considers the asymmetry of the energy peaks [17]. 

### 2.3. Experimental Set-Up

**Figure 7:**
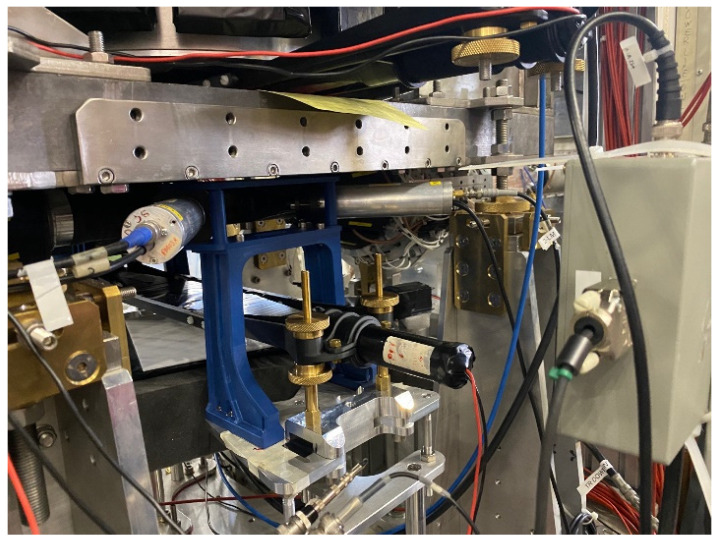
The experimental set-up at the DAΦNE collider at the INFN National Laboratories of Frascati (Italy). The gray box on the right side represents the detector box with the preamplifiers. The PMTs of the SIDDHARTA-2 luminosity monitor are clearly visible.

The spectroscopic response of the detector was measured at the laboratory of ionizing radiation detectors at the DiFC of University of Palermo (Italy). The detector was irradiated through the cathode electrode with uncollimated radiation sources (main X-ray and gamma lines: ^109^Cd, 22.1, 24.9, and 88.05 keV; ^241^Am, 59.5 keV; ^57^Co, 122.1 and 136.5 keV; ^137^Cs, 661.7 keV). We used two different ^241^Am sources: one emitting the Np L X-ray lines (13–21 keV) and the 26.3 keV gamma line, and the other one with these lines shielded by the source capsule. We used encapsulated radiation sources with a small active part inside a cylindrical steel capsule (diameter: 8 mm; height: 5 mm). To obtain the same ICR (input counting rate) of the impinging photons on the detector (through the cathode surface), we changed the solid angle subtended by the detector, i.e., the distance from each source to the detector. All measurements were performed at room temperature (T = 20 °C). During measurements at the DAΦNE collider, the detector was installed near the interaction point (IP) of the collider, vertically aligned with the SIDDHARTA-2 luminosity monitor (LM) [58], at a distance of 43 cm (Figure 7). The digital electronic device was installed outside the DAΦNE collider in the SIDDHARTA-2 control room (Figure 5). 

We performed *off-line* reduction of the background by also acquiring signals from the LM as detector channels. The LM signals were processed by a time-to-amplitude converter (TAC) module (ORTEC mod. 566). The LM, through the time-of-flight technique (TOF), allows the discrimination between kaons and the minimum ionizing particles (MIPs) produced during the e+ e− collisions [58]. The measurements at the DAΦNE collider were performed within a total acquisition time of 72 h, during which the accelerator delivered e− and e+ beams with average currents of 500 and 270 mA, respectively.

## 3. Measurements and Results

### 3.1. Spectroscopic Response of the Detector

**Figure 8:**
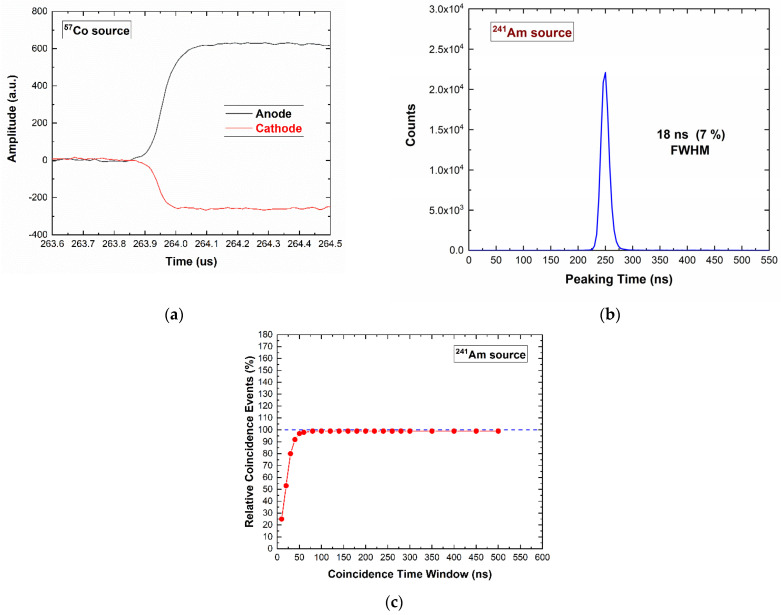
(**a**) The CSP output pulses from the detector–preamplifier at 122 keV (^57^Co source): anode (black line) and cathode (red line) electrodes. (**b**) Peaking time distribution of the pulses from ^241^Am source. (**c**) Coincidence events between the anode and cathode at different coincidence time windows.

Figure 8a shows the typical shapes of the detector–preamplifier output pulses (CSP output pulses) from both the anode (black line) and cathode (red line) electrodes of the detector. The pulses were characterized by fast leading edges with average peaking time of about 250 ns at −1000 V (negative bias voltage at the cathode). This was due to the behavior of the weighting field (Figure 2), which allowed the formation of pulses near the anode. In this case, the peaking time (proportional to the collection time of electrons) was lower than the expected one considering the thickness of the detector. The difference between the pulse amplitudes (or pulse heights) was due to the different gains of the two preamplifiers. The distribution of the peaking time values (^241^Am source), shown in Figure 8b, was characterized by a good time resolution of 18 ns FWHM. The coincidence events between the anode and cathode electrodes were fully detected within 50 ns, as shown in Figure 8c. 

**Figure 9:**
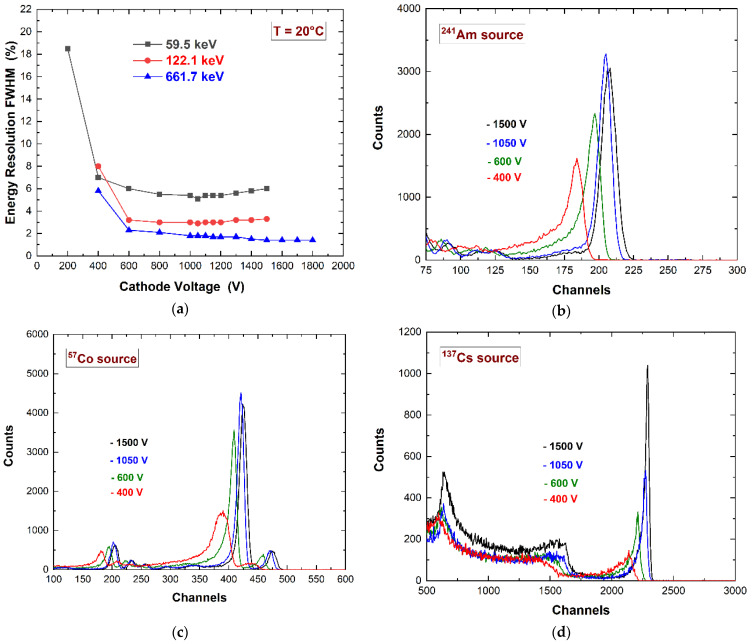
(**a**) Measured energy resolution (FWHM) vs. the cathode voltage. The energy spectra of (**b**) ^241^Am, (**c**) ^57^Co, and (**d**) ^137^Cs sources at different cathode voltages.

The spectroscopic performance of the detector was investigated at room temperature (T = 20 °C) using uncollimated radiation sources (^109^Cd, ^241^Am, ^57^Co, and ^137^Cs). Figure 9a shows the energy resolution (FWHM) vs. the cathode bias voltage measured at various photon energies. The energy spectra of ^241^Am (Figure 9b), ^57^Co (Figure 9c), and ^137^Cs (Figure 9d) sources at different cathode voltages are also presented, highlighting the improvements in charge collection efficiency. At low energies (^241^Am and ^57^Co sources), the best energy resolution was obtained at −1050 V as the best compromise between the collection and electronic noise contribution. At 662 keV, the energy resolution continued to improve up to −1800 V. This was related to the increased collection noise (incomplete charge collection) at high energies due to photon interaction near the pixel anode, which therefore produced pulses with high hole contribution [16,53]. In this work, we preferred to use the voltage of −1050 V in order to optimize the low energy range (<200 keV). 

**Figure 10:**
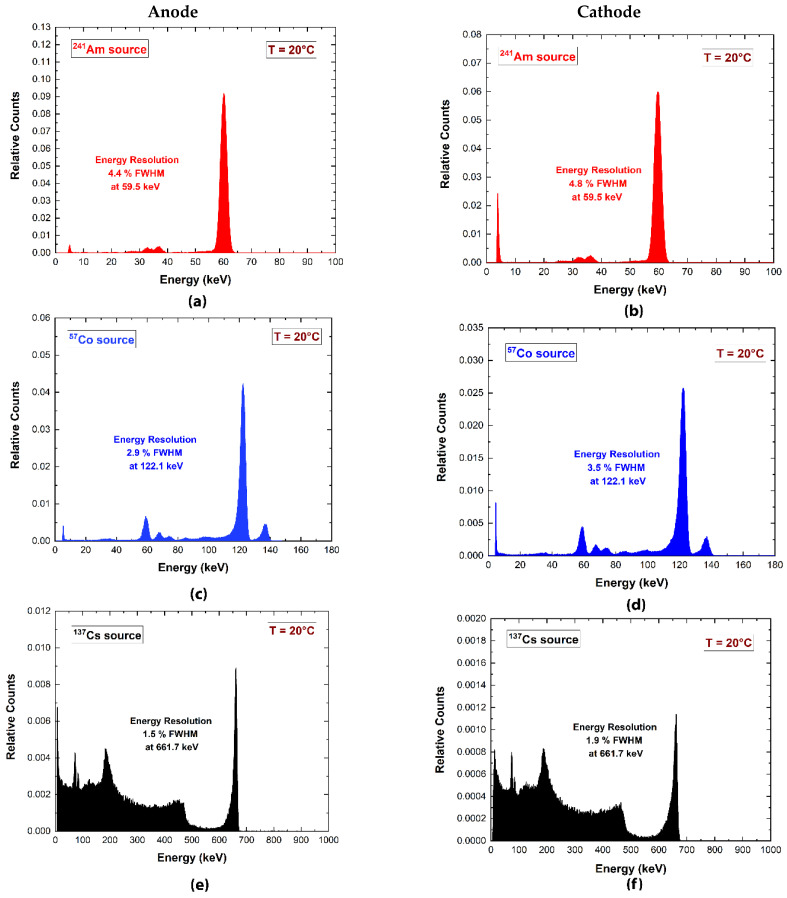
Measured (**a**) ^241^Am, (**c**) ^57^Co, (**e**) ^137^Cs energy spectra from the anode electrode and (**b**,**d**,**f**) from the cathode electrode. (**a**,**b**) The expected Np-L lines (L_α1_ = 13.9 keV, L_β1_ = 17.8 keV, and L_γ1_ = 20.8 keV) are shielded by the capsule of the ^241^Am source. (**c**,**d**) The W-K lines and Pb-K lines emitted by the ^57^Co capsule and the shielding set-up are clearly visible.

Figure 10 shows the energy spectra from the anode and the cathode electrodes, respectively.

**Figure 11:**
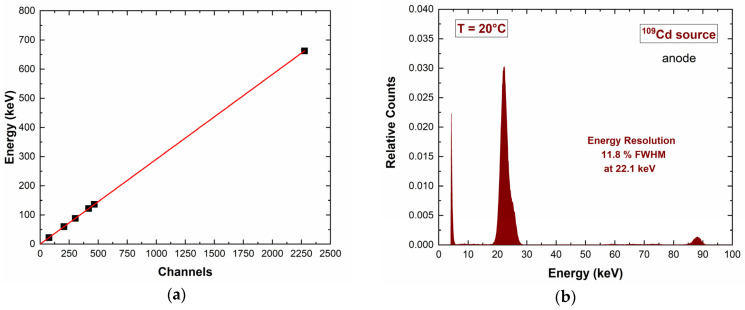
(**a**) Linearity of the detector response (anode pixel) with energy. (**b**) Measured ^109^Cd energy spectrum: the energy resolution does not allow a clear separation of the 22.1 and 24.5 keV Ag-K lines.

**Figure 12:**
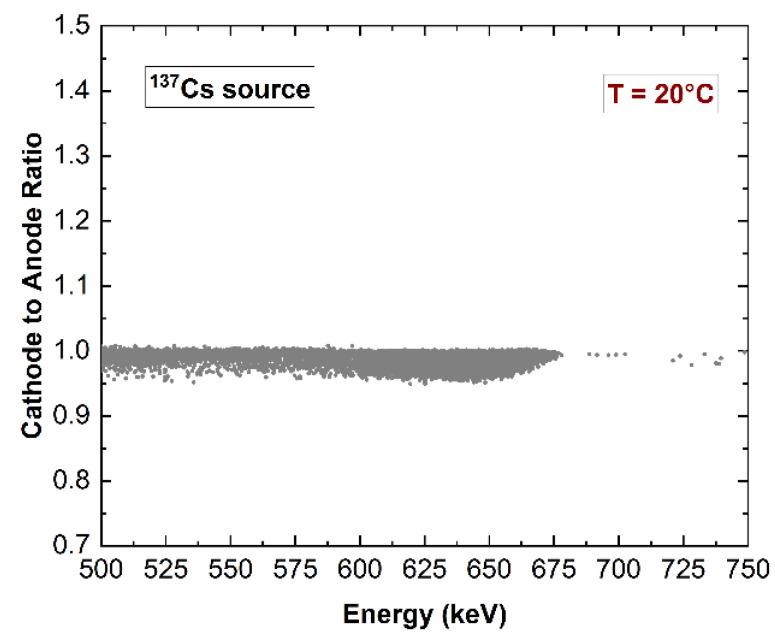
Scatter plot of the cathode-to-anode (C/A) ratio vs. the energy of the pulses from the anode (pixel).

The detector was characterized by a low detection energy threshold of 6 keV. The low-energy spectra (^241^Am source) were characterized by symmetric energy peaks with no hole tailing, highlighting the electron-sensing properties of the hemispherical electrode layout. Tailing was observed at high energy (^57^Co and ^137^Cs sources), i.e., for photon interactions near the pixel anode. The linearity of the detector response with energy was verified, as shown in Figure 11, where the ^109^Cd spectrum is also presented (Figure 11b). Due to the hemispherical electrode configuration, the cathode energy spectra were also quite good and similar to the anode ones. Indeed, the cathode-to-anode ratio (C/A), typically used for spectral improvements in CZT detectors [59,60,61,62,63,64,65], was independent from the anode energy (Figure 12). This demonstrates that a measurement of temporal coincidences between the anode and the cathode pulses is not necessary for performance improvements. 

**Figure 13:**
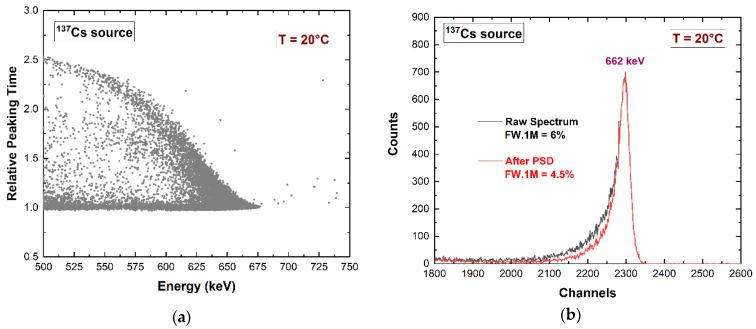
(**a**) Scatter plot of the relative peaking time vs. the energy of the anode (pixel). (**b**) Measured energy spectra: raw and improved spectra after pulse shape discrimination (PSD).

**Figure 14:**
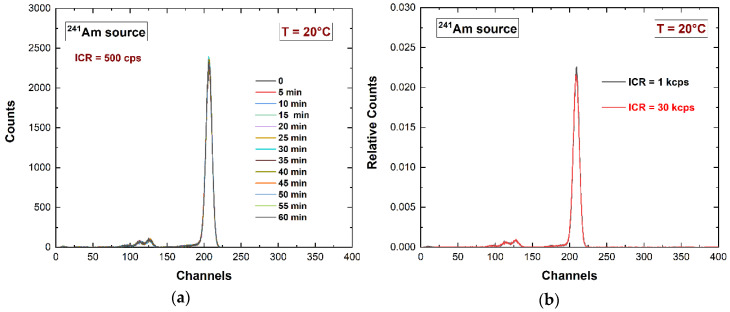
(**a**) Temporal stability of the spectroscopic response system up to 1 h. (**b**) Reproducibility of the energy spectra at different input counting rates (ICRs).

However, a small performance enhancement could be obtained through the pulse shape analysis of the pixel pulses, i.e., by analyzing the relationship between the peaking time and the amplitude (energy or height) of the anode pulses. The scatter plot in Figure 13a points out a reduction of the anode energy within the 662 keV energy peak (^137^Cs source) at long peaking times (relative peaking times > 1). This demonstrates that pulses with longer peaking times are characterized by an increased hole contribution and trapping. By applying a classical pulse shape discrimination (PSD) technique (Figure 13b), we obtained a reduction in the hole tailing in the 662 keV peak without rejecting events in the symmetry region of the main peak. The reduction of the peak asymmetry (hole tailing) was quantified by the reduction in the FW.1M [66]. The temporal stability of the detector was verified up to 1 h, as shown in Figure 14a. Moreover, Figure 14b shows the reproducibility of the spectroscopic response at different input counting rates (ICRs). This result is mainly due to the high-rate capabilities of the digital PSHA.

### 3.2. Measurements at the DAϕNE Collider

**Figure 15:**
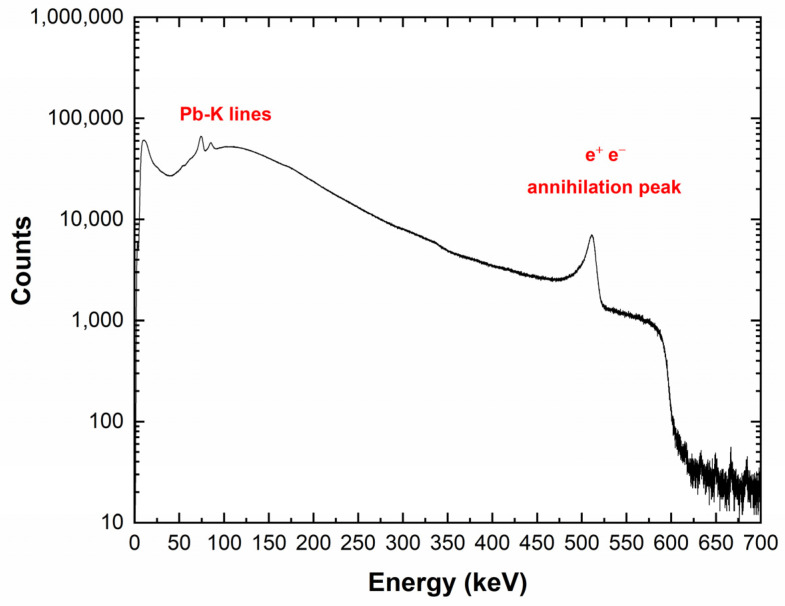
Energy spectrum measured with the CZT quasi-hemispherical detector at the DAϕNE collider (72 h). The Pb-K lines (75 and 85 keV) from lead shielding and the 511 keV annihilation peak are clearly visible.

**Figure 16:**
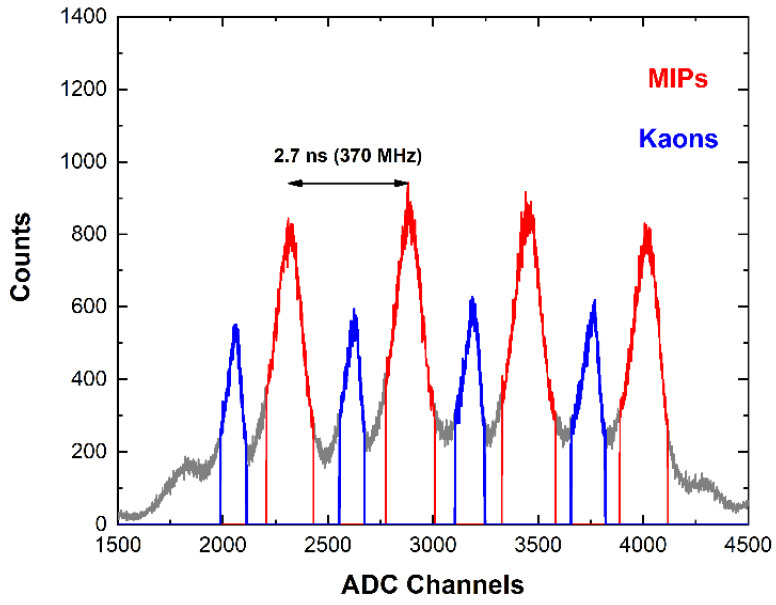
The spectrum from the TAC luminometers (LMs) of the SIDDHARTA-2 experiment. The four red peaks represent the arrival times of MIP events, while the blue peaks show the arrival times of kaons.

**Figure 17:**
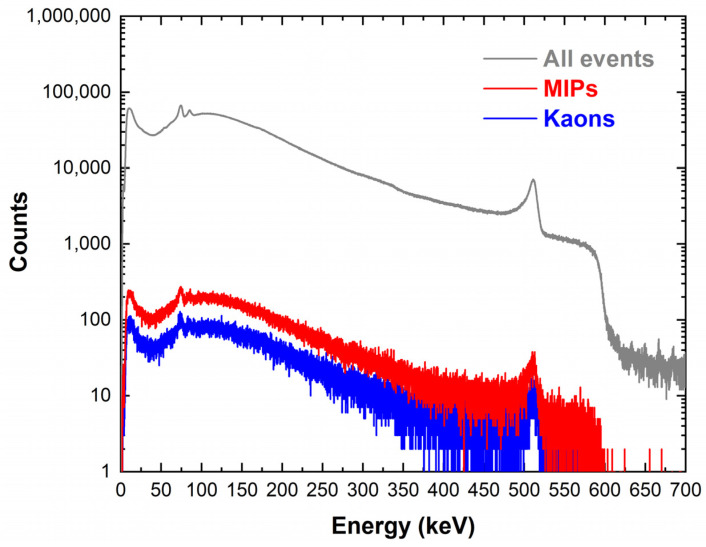
The energy spectra measured at the DAΦNE collider with no event selection (gray line; 1.45 × 10^8^ events) by selecting events in temporal coincidence with kaons (blue line; 2.3 × 10^5^ events) or MIPs (red line; 5.5 × 10^5^ events).

Figure 15 shows the energy spectrum measured with the CZT detector at the DAϕNE collider within an acquisition time of 72 h. The measured input counting rates ranged between 3 and 4 kcps. The Pb-K lines (75 and 85 keV) from SIDDHARTA-2 lead shielding and the 511 keV annihilation peak were clearly visible. To reduce the background events, we also acquired the events from the LM-TAC. The TAC signals were used to select only events related to a K+ K− pair passed through the two scintillators of the LM [9]. Figure 16 shows the TAC spectrum, where the blue and red peaks are related to kaons and MIPs, respectively. The LM scintillators produced two peaks due to the different arrival times between kaons and MIPs. The 370 MHz radio frequency (RF) of the DAϕNE collider was used in temporal coincidence with the signals from the LM to obtain a few ps resolution timestamp for each event. To reduce the frequency, an RF/4 was used at a frequency of about 90 MHz. Therefore, every coincidence event in the LM discriminators could be randomly associated in time with one of the four collisions; this is reflected by the four double structures of Figure 16. The energy spectra after selection for MIPs and kaons, i.e., the events in the temporal coincidence with the TAC signals (coincidence time window of 2 ms), are shown in Figure 17. The three energy spectra showed similar shapes, reflecting the absence, in this preliminary test, of a target where kaonic atoms could be formed. By considering the coincidence with K^-^ events, a rejection factor of 10^3^ was obtained.

**Figure 18:**
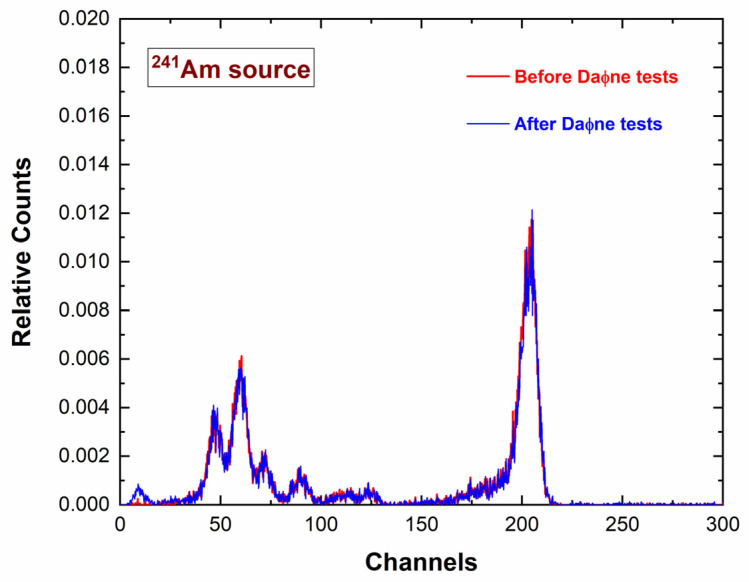
^241^Am energy measured before (red line) and after (blue line) the tests at the DAϕNE collider. In this case, we used a new ^241^Am source allowing the emission of the Np-L lines (L_α1_ = 13.9 keV, L_β1_ = 17.8 keV, and L_γ1_ = 20.8 keV) and the 26.3 keV gamma-ray line.

We also verified the reproducibility of the performance of the detection system and the absence of radiation damage by measuring and comparing the energy spectra before and after the tests at the DAΦNE collider, as shown in Figure 18. 

## 4. Discussion and Conclusions

The potential of CZT detectors for high-resolution measurements of hard X-rays from intermediate kaonic atoms are presented in this work. We developed a demonstrator prototype based on a new CZT quasi-hemispherical detector and custom digital pulse processing electronics with high timing resolution (<10 ns). Despite the quasi-hemispherical electrode layout being a consolidated geometry for CZT gamma-ray detectors, the proposed detector, fabricated using improved electrical contacts over the current state of art of CZT detectors, allowed us to obtain interesting key results:

- Low room temperature leakage currents (<1 nA at 1000 V) were measured, confirming the high quality of the fabricated detectors for optimal matching with low-noise charge-sensitive preamplifiers.
- The pixel size (750 µm diameter), properly selected after simulation, represents the best compromise between the reduction of electronic noise and the enhancements of the charge collection efficiency. In this study, it allowed interesting room-temperature energy resolution: 4.4% FWHM (2.6 keV), 3% (3.7 keV), and 1.4% (9.3 keV) at 59.5, 122.1, and 662 keV, respectively, overcoming the spectroscopic limits of these detectors on X-ray detection in the low energy range (<100 keV).
- The CZT module ensured a large detection area (10 × 10 mm^2^) with a single readout channel; moreover, further spectroscopic improvements could be achieved by digital pulse processing analysis (pulse shape analysis through the peaking times of the pulses) without the use of further detector channels.
- Successful tests within the DAΦNE collider environment were obtained, with particular focus on background reduction and the absence of radiation damage. The obtained background rejection factors of 10^3^ represent a promising result, which encourages us to include these detectors in measurements of radiative transitions from several intermediate and high-mass kaonic atoms.

Ongoing activities are focused on the development of a large area array (40 × 40 mm^2^) based on several CZT quasi-hemispherical modules for kaonic atom X-ray detection at the DAΦNE collider. Moreover, research activities will concern the development of low-noise charge-sensitive preamplifiers able to further improve the energy resolution of detectors at low energies. 

## Data Availability

Not applicable.
